# The Engineering Side of Hospital Work

**Published:** 1905-04-29

**Authors:** 


### HOSPITAL ADMINISTRATION.

### CONSTRUCTION AND ECONOMICS.

### THE ENGINEERING SIDE OF HOSPITAL WORK.

*Continued from page 76.*

X.—LAUNDRIES.—FINISHING.

The writer has grouped the various processes of blueing, starching, ironing, and getting up generally under the above title. The object of all of them is practically the same—to present the clothes smooth, and where it is required, as in nurses' aprons, caps, collars, etc., with the beautiful white gloss that is so much admired. Everyone who has slept at a country inn will remember the uncomfortable feeling presented by the rough dry sheets that are so often given the traveller to sleep between, and everyone is familiar with the fact that almost every article that goes to the wash looks better, and keeps clean longer, if it has a nice finish. Washing and drying, however done, leave the clothes in a rough state, with the particles representing the outer surfaces all apparently turned outward, making a very uncomfortable surface where they have to be worn next to the skin, and so arranged that each individual particle appears in the best possible condition for attracting particles of dust, etc., that are flying about or with which they come into contact. The old-time process consisted of soaking the articles in blue, or in starch, and then ironing or mangling them, according to their use. The same process holds in the machine laundry, but it is aided at every step by machines arranged to perform the different operations on several things at the same time, or if individually, very quickly. There are starching and blueing machines of various designs arranged to not only soak the starch into the clothes, but to rub it in. There are machines also for preparing the starch, something on the lines of the apparatus for preparing the soap liquor. In Messrs. Braithwaite's apparatus for preparing the starch there is a copper vessel carried inside another vessel of galvanised iron, the space between being packed with material of high thermal resistance. The starch is heated by a jet of steam, and the idea is to enable any starch that has been prepared and has not been used to be kept till required, the jacket of heat-resisting material preventing the egress of the heat, and so preserving the starch in usable condition. Messrs. Bradford make an apparatus consisting of a copper kettle with a jacket in which steam is allowed to pass. In either case, and in the other apparatus made by the different makers, the starch is prepared for use with the minimum of waste, and in the best form for application to the clothes.

After starching comes ironing. In every laundry there are still a good many of the apparatus called in country districts "flats" in use. While machinery will always assist very materially any operation in which a large number of things have to be produced, or to be handled, where the number is small machinery is sometimes wasteful. And in all laundry work there are usually some things that are not adapted for machining. In pretty well every industry the same thing occurs. The bulk of the work is done by machines, but there are nearly always some things that have to be done by hand tools. Hence the presence of the flat-iron. Machinery, however, comes in to assist in the process of heating the irons. Special stoves are constructed, some in pyramidal form, with steps upon which rows of irons rest while heating, and others in cylindrical and other forms, designed to accommodate a number of irons at the same time, the stoves being heated with coke, anthracite coal, or by gas. The individual irons are also arranged to be heated by gas, the latest arrangement being what is termed the "blast" system, in which air is supplied to the irons under pressure, so that the combustion of the gas may be complete, thereby avoiding the noxious fumes that are present where incomplete combustion takes place, and giving a distinct economy in the quantity of gas consumed. Both gas and coal require a certain definite quantity of oxygen, which is provided by a certain quantity of atmospheric air for the complete combustion of every pound of coal, or coke, and for every cubic foot of gas; and when the gas or the coal receives and uses this quantity of oxygen, the largest possible amount of heat is given out in the process of combustion. It is never possible to obtain theoretical perfection in engineering, but the nearer to theoretical perfection the engineer can go, usually the cheaper the work is done. The blast of air is provided by means of fans, as will be explained when dealing with ventilation. A current of air is directed into a pipe which has a connection with the burner of the gas iron, or stove. The burners for gas irons are the usual well-known type, in which the gas is allowed to mix with a certain proportion of air before passing to the jets, where it is consumed. In the modern gas iron the proportion of gas and air can be regulated—a matter of some importance, as gas itself varies very considerably, sometimes from hour to hour. Th process of gas-making is a somewhat complicated one. Since gas companies have had to meet the competition of electricity, every effort has to be made to cheapen production, and the working of the gas-producing plant may easily vary, according to the supply of different materials. In addition, gas-pipes leak, and air leaks into the pipes by the same way as the gas leaks out, and this is particularly the case where the gas has to be delivered at high levels.

For things of which there are a great many of the same kind, such as sheets, nurses' skirts, etc., ironing machines of various sizes have been designed. First, however, another machine comes in to assist, in the dampener and shaker. Clothes require slightly moistening before ironing, and it saves time in preparing them for the ironing machine to have them loosened after they come out of the centrifugal machine. As will easily be understood, the clothes press very close together in the process of extracting the water in the centrifugal machine, the force applied being considerable. The ironing machines all consist of one or more hollow cylinders of iron or steel, highly polished, bedding in hollow frames, semi-cylindrical in shape, the beds being hollow, and arranged to receive steam or the mixture of gas and air mentioned above, in connection with gas irons. The rollers or cylinders are usually covered with felt or some similar substance. Here, it will be seen, are all the components of the old-time washerwoman's ironing apparatus; the bed corresponding to the iron, the roller, with its covering, to the ironing-board, with its blanket. The sizes and general arrangement of the ironing machines depend entirely upon the articles to be handled. Perhaps one of the most striking sights in a modern laundry, or, for that matter, anywhere, is a large Decoudun ironing machine at work. It consists of a hollow cylinder sometimes as long as 12½ feet, and is intended for sheets and other large articles. It is also made in smaller sizes, down to 4 feet 6 inches long. The large rollers are 3 feet 6 inches in diameter, and the others proportionally smaller, the 4 feet 6 inch rollers being 14 inches in diameter. One or two attendants stand on each side of the roller, the attendants on one side feeding the clothes to the machine and those on the other side receiving them as they emerge ironed. Messrs. Manlove, Alliott and Co. provide guards to the apparatus which prevent the fingers of the attendants being caught as they feed the clothes to the machine. They also arrange to heat both the beds and the rollers by steam or by gas, the advantage of this being, they claim, that the clothes to be ironed can be taken to the ironing machine directly from the centrifugal wringing machine, instead of passing through the drying-rooms—a matter of considerable saving in time and cost. Messrs· Braithwaite make ironing machines of the same type, with as many as five rollers all lying in their own beds, the beds only being heated. The clothes in these machines pass round the different rollers in succession, the clothes being subject to heat and smoothing at each roller. The rollers are driven by power from any convenient source, the most common being a belt from a countershaft passing either overhead or in a well in the ground, the shaft receiving power from the engine driving the whole of the laundry and distributing it where it is required. Each machine has its driving arrangement, consisting of two pulleys, one of which runs loose on its shaft and is in gear when the machine is not running, the other being connected to the driving-shaft of the machine itself, and being thrown into gear when the machine is to be run. A fork, provided with a handle easily reached, throws the strap from one pulley to the other, and so delivers the power to the machine, or cuts it off, as required. The gearing should be all fenced in. In modern laundries the tendency is more and more to drive every machine taking any quantity of power by its own electric motor, which is geared to the machine in any convenient way, usually by what is termed spur, or wheel-and-pinion gearing, a small pinion wheel gearing into a larger wheel. The motor is started and stopped at will by a switch fixed in any convenient position. Where there are a group of machines together they are often driven by one electric motor through a small countershaft, the arrangement for delivering the power to each machine being then the same as where the whole laundry is driven by an engine. In addition to the smaller sizes of the Decoudun machine there are several machines constructed on precisely the same lines, with one or more rollers and a bed, but in which the arrangement of the bed, and sometimes of the rollers, is different from that of the large ironing machines described above. Thus, some machines have a small table, on which the garment is laid, while a heated roller passes over it, or the table may move under the roller. In others there are two rollers hanging free from the machine, not supported at both ends, as with the other machines, the garment to be ironed being slipped in between the two. Again, there are machines arranged to take neck-bands, and to iron them on both sides at the same time, and very quickly. Messrs. Braithwaite also make an apparatus for finishing woollen garments that require to be finished with the nap raised. It consists of a hollow shelf, supported upon an iron standard. The top of the shelf is perforated with a number of fine holes. On the perforated shelf a piece of open-meshed fabric is laid, and the garment to be finished is laid on the top of the fabric, dry steam being then turned on in the inside of the hollow shelf. The result, it is stated, is, the nap rises in the manner required. Again, there are goffering machines, consisting of pairs of fluted rollers, gearing into each other, the garment passing between the rollers and receiving the impress of the flutings. There are also irons of various patterns to fit different garments, such as caps, sleeves, etc. And, again, there is the humble mangle, very much as Mr. Mantalini may have turned it, but made to turn by power, and of more lasting materials. The mangle, it will be remembered, is merely another of the apparatus esigned to smooth out the roughnesses left in clothes as they come from the drying-room, and is intended for those things that do not require a high finish.

In the next article the writer will describe the laundry arrangements in use at the principal hospitals in London.

